# A new grounded theory model of sexual adjustment to HIV: facilitators of sexual adjustment and recommendations for clinical practice

**DOI:** 10.1186/s12879-019-4727-3

**Published:** 2020-01-13

**Authors:** Ben Huntingdon, Louise Sharpe, John de Wit, Martin Duracinsky, Ilona Juraskova

**Affiliations:** 1 Clinical Psychology Unit, School of Psychology, The University of Sydney, Brennan MacCallum Building (A18), NSW, Sydney, Australia; 2 Centre for Social Research in Health, UNSW Sydney, Kensington, NSW Australia; 3 Department of Interdisciplinary Social Science, Utrecht University, Utrecht, The Netherlands; 4 Sorbonne Paris Cité, EA 7334, Patient-Centered Outcomes Research, Université Paris-Diderot, Paris, France; 5 Service de Médecine Interne et d’Immunologie Clinique, Hopital Bicetre, Kremlin-Bicetre, France; 6 Unité de Recherche Clinique (URC-ECO), hopital Hotel-Dieu, Paris, France; 7 Centre for Medical Psychology and Evidence-based Decision-making (CeMPED), The University of Sydney, Sydney, Australia

**Keywords:** HIV, Sexual wellbeing, Grounded theory, Qualitative research, Adjustment model, Sexual behaviour

## Abstract

**Background:**

Life expectancy of people living with HIV (PLWH) is increasing. Effective biomedical prevention methods (treatment as prevention and preexposure prophylaxis) are being widely implemented in high-income nations. Therefore, research into quality of life, including sexual adjustment, is of increasing importance to HIV care. Yet, sexual adjustment of PLWH has been neglected in past research. We propose a new model of sexual adjustment to HIV which explores the dynamic process, facilitators and barriers characterising sexual life of PLWH overtime.

**Method:**

Thirty PLWH (19 male, 11 female) recruited from two HIV treatment centres as well as community groups, completed semi-structured interviews which were audio-recorded and transcribed verbatim for analysis using grounded theory.

**Results:**

The model of sexual adjustment to HIV is the first to establish how undue fears of transmission of HIV during sex and/or fear of rejection by sexual partners determine initial sexual behaviour after diagnosis and also sexual adjustment over time. Within the model, sexual adjustment to HIV is facilitated by factors which assist PLWH to overcome such fears, including: partner acceptance, peer, community and health professional support, and accurate knowledge of risk of transmission including of undetectable viral load and pre-exposure prophylaxis. Adjustment is inhibited when undue fears of transmission and of rejection persist long term, resulting in maladaptive behaviours to cope with such fears including avoidance of sex and problematic drug and alcohol use.

**Conclusion:**

This model offers clear directions for promoting sexual adjustment to HIV. Health professionals should: (a) assess and intervene for sexual quality of life (not just risk) among PLWH; (b) be aware that serosorting facilitates adjustment in the short to medium term, but may interfere with adjustment long-term, (c) promote opportunities for positive connection between PLWH, and (d) intervene directly with PLWH and HIV negative sexual partners to promote accurate risk of transmission knowledge, including how this applies to their own sexual practices, and whether they are experiencing undue fear of transmission over time.

## Background

With early diagnosis and treatment commencement, the life expectancy of people living with HIV (PLWH) is approaching equivalence with that of the general population [1, 2]. Therefore, living well with HIV long-term, and sexual adjustment in particular, are becoming increasingly important issues in HIV care. Qualitative research methods offer a fitting means of studying sexual adjustment as they focus on experiences and processes behind phenomena of interest [3]. However, a recent thematic synthesis of qualitative research on sexual life with HIV found the topics of condom use and disclosure of HIV positive status to be dominant in past research [4], with little information provided on the processes inherent in sexual adjustment to HIV with which to promote sexual wellbeing rather than mitigate sexual risk.

The need for research into sexual adjustment of PLWH is particularly pressing given that strong support has recently been established that the risk of transferring HIV during sex with consistent undetectable viral load (UDVL) is effectively zero [5–7], a message which has been shortened to undetectable = untransmitable (U=U) [8]. Coupled with the increasing use of pre-exposure prophylaxis (PrEP) by HIV negative sexual partners of PLWH [9–11], these developments mean that earlier research on the impact on sexual functioning and adjustment following HIV diagnosis may no longer be applicable. Feminist and sexual rights perspectives have been applied to theorise ways to promote the sexual well-being of women with HIV [12]. Yet, there is currently no model of factors which promote sexual adjustment to HIV to guide further research and intervention into this important aspect of quality of life for PLWH. To address this gap, the current qualitative study therefore aimed to explore the process of sexual adjustment to HIV, and propose a new grounded theory derived process model of factors affecting sexual life over time.

## Method

Charmaz’s (2016) constructivist variant of grounded theory qualitative research was selected due to its focus on analysing processes, and the use of inductive methods to build theory of such processes. Constructivism holds that there is no objective reality separate to human understanding [3].

### Participants

PLWH were recruited through counselling teams at two public HIV clinics in Sydney, as well as through community groups to ensure representation of those seeking psychosocial support at the time of the interview and those who were not. Inclusion criteria were: 18 years or older, a diagnosis of HIV and living in Australia. Exclusion criteria included: insufficient English language ability to participant in a qualitative interview or complete a written questionnaire, current psychiatric or neurocognitive disorder which would impede participation.

### Procedure

Consenting participants initially completed a demographic questionnaire, the Depression Anxiety Stress Scales short form (DASS-21) [13], and the PROMIS v2 sexual satisfaction questionnaire [14] to characterise the sample, before participating in a semi-structured telephone interview. See Table 1 for the interview guide used. Interviews were audio recorded and transcribed verbatim for analysis. To ensure representation of key aspects of the process of sexual adjustment, a process of preliminary and iterative analysis was used to select further participants for interview until data saturation was reached (i.e. theoretical sampling) [15]. Audio and written data were de-identified prior to analysis.

**Table 1:** Semi-structured interview guide

Order of questions asked	
1. Would you tell me about the time in your life when you first found out you had HIV?	
2. What did (or has) treatment for HIV involve(d) for you?	
3. How do you feel, generally speaking, at this time? *[For questions 4–7 – prompt for type of sexual behaviour, practices and satisfaction, whether this changed or stayed the same over time, reasons for changes or staying the same]*	
4. How would you describe your sexual life before you were diagnosed?	
5. How would you describe your sexual life just after you were diagnosed?	
6. [If applies] how would you describe your sexual life when you started having treatment?	
7. [If different from above] how would you describe your sexual life in the past 4 weeks?	
8. Has living with HIV influenced your romantic or sexual relationship/s? If so, tell me more about that.	
9. Has the way you interact with current, past or potential sexual partners changed or stayed the same since being diagnosed? If so, how? Tell me more about that.	
10. How do you feel about the way that your treatment team have addressed sexuality in the context of HIV?	

### Data analysis

One author (BH) led the grounded theory analysis, with cross coding and discussions of emerging codes and themes conducted by a trained research assistant and two other authors (IJ and LS). The analysis process included: 1. initial free coding of action and processes, 2. focused coding using most frequent and important codes to synthesise data, and 3. axial coding by defining the situations, actions taken and consequences of such actions by participants as they navigated their sexual lives [16]. Constant comparison between and within participants in similar contexts or engaged in similar actions was used to build themes which describe the process [17].

To enhance trustworthiness and credibility an audit trail was created by free coding directly onto interview transcripts, and then saving each interim stage of the focused and axial coding before continuing on to the next. Blind independent cross-coding was conducted by a second qualitative researcher on a sub-set of interviews and compared to the lead author’s coding to reduce bias. In addition, three authors (BH, LS, IJ) collaborated on the analysis and interpretation of quotes. Moreover, the lead researcher kept an analytic diary to track their assumptions, preconceptions and values, to ensure that these did not unduly influence the analysis. Regarding reflexivity, the lead author who conducted all interviews had no pre-existing or ongoing relationship with the participants.

## Results

Thirty participants (19 male, 11 female) out of 45 PLWH who agreed to be contacted completed the interview and questionnaire. Average interview time was 49.4 min (SD = 10.8). Demographic and clinical sample characteristics are provided in Tables 2 and 3, respectively. Years since diagnosis ranged from 1 to 30 (M = 10, SD = 8.4). Psychological distress scores were in normal ranges for 8 participants, in mild to moderate ranges for 12 participants and in severe to extremely severe ranges for 10 participants. Sexual wellbeing scores were average or above average for 43.2% of the sample.

**Table 2:** Participant demographic information (*n* = 30)

	Mean (SD)
Age	42.4 (11.3)
	n (%)
Gender	
Female	11 (36.7)
Male	19 (63.3)
Marital status	
Single	17 (56.7)
Married / de facto	11 (36.7)
Separated / divorced	1 (3.3)
Widowed	1 (3.3)
Employment	
Employed	19 (63.3)
Student	2 (6.7)
Home duties	3 (10)
Retired	2 (6.7)
Unemployed	4 (13.3)
Highest Education	
Some of high school	4 (13.3)
All of high school	3 (10)
Tertiary certificate or diploma	9 (30)
Tertiary degree or post graduate degree	14 (46.7)
Sexual orientation	
Heterosexual	10 (33. 3̇)
Homosexual	19 (63. 3̇)
Bisexual	0
Identify with none of these	1 (3. 3̇)
Place of birth	
Australia	16 (53.3)
North America	2 (6.7)
South America	1 (3.3)
Pacific	2 (6.7)
UK & Europe	3 (10)
Asia	4 (13.3)
Africa	2 (6.7)
Language spoken at home	
English	29 (96.7)
Language other than English	1 (3. 3)

**Table 3:** Participant clinical information (n = 30)

HIV related information	Mean (SD), [range]
Years since HIV diagnosis	10 (8.4), [1–30]
Years since HIV treatment commenced	7.6 (7.5), [1–26]
	n (%)
Mode of HIV acquisition	
Sexual transmission	29 (96.7)
Not known	1 (3.3)
Currently on HIV treatment	26 (86.7)
Self-reported viral load	
Undetectable	26 (86.7)
Detectable	1 (3.3)
Unknown	3 (10)
Self-reported CD4 count	
500–1500 range	21 (70)
200–400	2 (6.7)
< 200	0 (0)
Unknown	7 (23.3)
Psychological well-being	Mean (SD), [range]
DASS^a^ sub-scale scores	
Depression	11.2 (8.7), [0–36]
Anxiety	7.8 (6.3), [0–24]
Stress	13.3 (8.8), [0–36]
Sexual well-being	n (%)
PROMISv2^±^ normed score ranges	
No partner/ No recent sexual activity	13 (43.3)
Below average (~ 1 SD below mean)	4 (13.3)
Average	4 (13.3)
Better than average (~ 1 SD above mean)	1 (3.3)
Much better than average (>1SD above mean	8 (26.6)

^a^Depression Anxiety Stress Scales – short form 21 [13]

±PROMISv2 [14]

### The process of sexual adjustment to HIV

A positive adjustment outcome was defined by sexual activity about which the participant was satisfied, while a negative adjustment outcome was defined by barriers to sexual activity about which the participant was dissatisfied. Participants’ reports of their sexual activity and satisfaction, in combination with their sexual activity and satisfaction as measured using the PROMIS scale [14], were used to form judgements of sexual adjustment or maladjustment.

### THEME 1: Reactions to HIV diagnosis

Immediate emotions felt at diagnosis were related to the year of diagnosis. Once these initial emotions faded, some combination of enduring fear of HIV transmission and/or fear of rejection by a sexual partner determined sexual behaviour in the initial post diagnosis period.

#### Theme 1.1: emotional reactions at diagnosis

In addition to the commonly experienced shock at diagnosis, for those diagnosed pre-combination therapy, most felt they had been given a death sentence, thus triggering prominent fear of death.

“*Something that played in my mind is that I would die …* .” ID:009, male, DX: late 90s

In contrast, among those diagnosed since 2010, once the initial shock settled, some felt relief. For a few this relief related to now knowing why they had been feeling unwell, while for others, the relief was associated with no longer being worried about acquiring HIV - since sex for these latter participants before diagnosis involved risk-taking which while enjoyable, created regret and fear of acquiring HIV.

*“My first response was obviously shock. The very next emotion... was relief, because then I knew what was wrong with me … Also, because my doctor reassured me that [HIV] is manageable and treatable.*” ID:035, female, DX: late 00s

“*When I knew I had [HIV], I didn't have to be worried about [acquiring] it anymore … Just relief at not having to fear it anymore.”* ID:002, male, DX: late 00s

#### Theme 1.2: initial sexual life after diagnosis

Three main behavioural responses characterised initial sexual life after diagnosis: i) sexual inactivity, ii) no interruption to sex life, and iii) increased frequency of sex. The majority of participants described ceasing their sex life at the point of diagnosis. They attributed this sexual inactivity to a combination of fear of transmission of HIV during sex, and/or fear of rejection by a sexual partner.

“*After diagnosis I was actually scared shitless to have sex with anyone. I thought: 'No. That's it, I'm never having sex with anybody ever again’*.” ID:035, female, DX: late 00s.

“*Fear of having to tell someone that you’re HIV-positive - I think that was the predominant factor [related to sexual inactivity] for me*.” ID:042, female, DX: early 10s.

Some participants reported no change in sexual behaviour following diagnosis. These participants emphasised normalcy in their sexual lives post-diagnosis which they attributed to having a regular partner/s and their acceptance.

“*It was good, we were just married, so we were having sex quite often. Just normal sex*.” ID:033, female, DX: early 10s

“*The people that I was having sex with at that time were all fine with [me having HIV]. Things continued as normal for a little while.*” ID:003, male, DX: early 00s

Other participants, all of whom were male, described having sex without condoms with other HIV positive people only (serosorting), and feeling now free of the fear of acquiring HIV. They described the frequency of sex increasing and boundaries of sexual practice expanding.

*“ … there was this exploration … [within my sex life] I didn't have before … It was a bit more exciting and I was being a bit more extreme and doing things [sexual practices] that I didn't do before [I had HIV].”* ID:002, male, DX: late 00s

### THEME 2: facilitators of sexual adjustment

A number of factors emerged which seem to facilitate sexual adjustment over time by assisting participants to overcome fears of transmission of HIV and/or fears of rejection, including: partner acceptance, serosorting, peer and community support, knowledge of transmission risk (e.g. UDVL and PrEP), and health professionals who actively promoted sexual wellbeing (rather than solely focusing on ‘risk of transmission’).

#### Theme 2.1: Partner acceptance mitigates fears

While partner acceptance had the potential to promote adjustment, rejection by a sexual partner interfered greatly with sexual adjustment.

*“ … I said I was HIV-positive, and without even missing a beat, he turned around and punched me in the face and started screaming at me: ‘Get away from me … ’”* ID:031, female, DX: mid 90s.

With their partner’s acceptance and support, some participants reported that their sex life continued on, uninterrupted by diagnosis. Others described an ongoing process of adjustment from the point of diagnosis, within which their partner’s acceptance assisted the HIV-positive person to accurately perceive and adjust to the idea of HIV transmission.

“*I think in any relationship between anyone that’s [HIV] positive and negative, it can pass on [the virus] … I think it was more emotional and it overruled the facts sometimes, so there was an anxiety about it … [my partner] played a big role in trying to make me feel comfortable … He hugged me more. It was tenderer … things that were reassuring me that everything was okay*.” ID:011, male, DX: early 00s

However, a partner did not facilitate adjustment simply by being willing to have sex or to continue the relationship, without acknowledging the HIV diagnosis. When sexual partners denied that HIV was now present in their sexual life, or when they ignored their partners’ fears of transmission, this hindered the adjustment process. For adjustment to occur, the partner had to communicate acceptance of HIV as a part of that person, and physically demonstrate no undue fear of acquiring HIV.

*“I was very scared of giving [HIV] to my husband. He wouldn’t use protection and he said he loved me and if he caught it so be it … I tried to avoid the situation … it just became an ingrained bad habit to try and avoid [sex].”* ID: 046, female, DX: mid 00s.

*“I told him [I was HIV positive] after our first date. I remember I couldn’t look at him so I turned away, and I just felt him lean across and put his head on my shoulder and say he didn’t care; he just wanted to be with me … It was the first time that someone accepted me sexually.”* ID: 031, female, DX: mid 90s.

If a partner was unduly fearful of transmission (based on risk of transmission knowledge available at the time) and conveyed this in their sexual behaviour by appearing scared, or taking unnecessary precautions against transmission (such as refusing to perform oral sex for fear of transmission), this interfered with adjustment. Conversely, if a partner had confidence in reasonable precautions against transfer of HIV without undue fear, this appeared to facilitate adjustment.

*“The first time, he was wearing two condoms … he was wearing gloves to touch me … I think he was scared and I think he still is scared about it … I was always feeling a little bit low because of that.”* ID: 048, female, DX: early 00s

“*He's never been scared or worried... that he could contract [HIV] … If he had reacted a different way and been scared about it, it would have completely changed everything …* ID: 033, female, DX: late 00s

#### Theme 2.2: Serosorting to avoid fears of transmission and rejection for a time

Many participants used serosorting as a way of re-engaging with sexual life after diagnosis, as it provided freedom from both difficulties with disclosing HIV positive status (avoiding fear of rejection) and fear of HIV transmission. Some then transitioned into having sex with HIV-negative people and worked through difficulties with disclosure, while others found long-term partners through serosorting.

*“[By serosorting] you don’t have to worry about disclosing [having HIV] … or even the possibility of infecting someone else … I remember when I was recently diagnosed, I was freaking out that even with the condom on, I would give someone HIV. But obviously it’s not the case when you’re undetectable...”* ID: 037, male, DX: mid 10s

*“It was easier for me to have a [HIV-]positive partner than to have a negative partner. I never even thought of having a [HIV-]negative partner … It just made it a lot easier than trying to cross that bridge with someone that you start having emotions for and then … how do you actually tell that person that you are positive?” ID: 035, female, DX: late 00s.*

After diagnosis, for some participants, all of whom were male, the frequency of sex increased and boundaries of sexual practice expanded, as they were serosorting and were now free of the fear of acquiring HIV. Such freedom created a new enjoyment of sexual life for some participants, yet others described losing track of their personal boundaries, which was commonly attributed to crystal methamphetamine use.

*“ … there was this [sexual] exploration that I could have I didn't have before … [my sexual life] was a bit more exciting and I was being a bit more extreme and doing [sexual practices] that I didn't do before [I had HIV].”* ID:002, male, DX: late 00s

*“After the diagnosis, I started to explore things that I would usually not do [in my sexual life] … I started to use meth which was something really new and for whatever it does to everyone. I guess I lost my limits.”* ID:014, male DX: late 00s

#### Theme 2.3: Peer and community support promotes self-acceptance

Participants reported that engaging with the HIV-positive community (both informally and through community group events) facilitated their sexual adjustment; describing the importance of connecting with others living with HIV, finding self-acceptance and role models by observing others, and sharing strength in the face of difficulties.

*“I got to meet other [HIV-]positive guys, especially the older ones and just sort of learnt from their experience … it helped with that self-acceptance of there’s a large proportion of people who are [HIV-]positive and they live a normal life, and they’re okay with it - so it’s almost like a reassurance I guess.”* ID: 037, male, DX: mid 10s

*“Particularly women, but also people living with HIV and AIDS … we kept supporting each other. I'm a strong believer in peer support... If I didn't have that, things might have been different - if I had have felt isolated*.” ID: 032, female, DX: mid 80s

#### Theme 2.4: Using HIV transmission knowledge, including UDVL and PrEP, to overcome fear

Even prior to the widespread dissemination of the undetectable = untransmittable (U=U) message since 2016, knowledge about transmission risk played an important role in sexual adjustment. Some described an accurate understanding of risk of transmission, which influenced their decision to temporarily cease sexual life. For example, sex life was resumed once undetectable viral load (UDVL) occurred. For others, despite an accurate understanding of risk of transmission, fear of transmission persisted and they felt unable to have sex. Even when their sex life recommenced, some described a constant preoccupation and intolerance of even a small possibility that HIV could be transmitted, which affected the quality of their sexual life.

“ *… then going on the medication it took another two to three months before it dropped down to being undetectable. I basically ‘touched’ nobody until I was undetectable.*” ID: 028, male, DX: early 10s

*“Nearly every time I would go and have sexual activity, it would be a constant fear, a constant thought that’s throughout the entire moment and time that I was with the other person - that [fear of transmission] was always on my mind. So, the [sexual] enjoyment factor was down..*.” ID: 041, male, DX: mid 00s.

By the time of the interview, almost all participants understood that having an UDVL meant the chances of transmitting HIV during sex were effectively zero. This knowledge appeared to reduce fears of transmission and rejection. In addition, the increasing use of pre-exposure prophylaxis (PrEP) by HIV-negative people has had a similar effect of reducing such transmission/rejection fears.

*“it does wonders for your mental health and knowing that you can’t transmit the virus … I was so terrified of transmitting [the virus] and too terrified to tell people I had HIV. [Having UDVL] has allowed me to not only tell people but it’s allowed me to truly, for the first time in my life, negotiate the type of sex that I want to be having*.” ID: 013, male, DX: late 00s

*“[My partner] is on the ‘thing’ that he won’t get it [PrEP]. My HIV load is undetectable and everything is completely fine anyway. We don’t really worry about [transmission].”* ID:033, female, DX: late 00s.

While the widespread dissemination of the U=U message has had a strong impact on sexual adjustment of some participants, others reported ongoing difficulties overcoming undue fear of transmission, despite accurately understanding the U=U message. For some participants this was noticeably because the information given by their doctors had changed as the evidence behind risk of transmission had changed.

*“I’m not going to lie. I still worry [about HIV transmission]. I’ve gone to workshops … and I’ve read about it a lot … even if I’m doing it the absolute safest way to have sex I still worry of that 1% that I could transmit it to someone. It’s a small number but it has a big effect on my sex life.”* ID: 018, male, DX: mid 10s.

*“it wasn’t until I came here to [treatment centre named] and the doctors were telling me the complete opposite of what the doctors in [previous location] has been telling me that “you’ve been undetectable for months now. Why are you still not back [to usual sexual activity?”* ID: 018, male, DX: mid 10s

#### Theme 2.5: Health professionals interested in sexual wellbeing not just risk

Participants referred to positive relationships with their health professionals, however, this alone appeared insufficient to promote sexual adjustment. Rather, from participants’ perspectives, health professionals facilitated sexual adjustment when they actively conveyed that they cared about the PLWH’s (as well as their partner/s’) sexual-wellbeing, and not just their sexual risk.

*“At the beginning [of a consultation] they say, ‘Okay. Is your partner [HIV-]positive or negative?’ ‘He is negative’. ‘Are you using condoms?’ ‘Yes’. Those kind of things, like more general stuff. I’ve never heard any doctor get into more details … I don’t think they want to know either [about my sex life] … just making sure I didn’t pass it on...”* ID: 044, male, DX: early 00s.

*“My first doctor in [location] was incredibly good; she asked me if I was having a healthy sex life. My partner … was quite hypersensitive about risk. She called him in and talked him through what was risky and what wasn’t. She said at one point that if she was ever giving me a rectal exam with a finger, and the glove broke, she said to him, ‘I would never start PEP then’ … just saying little things like that to him was enough and really helped, just from a doctor’s point of view. She really went out of her way...”* ID: 013, male, DX: late 00s.

### THEME 3: barriers to sexual re-adjustment

Some participants described difficulties with sexual re-adjustment which appeared to be due to the absence of the supportive peers, partners, or health professionals, or because of difficulties with understanding or acting upon UDVL or PrEP knowledge described above. As a result, these participants continued to have undue fear of transmission of HIV, and strong fear of rejection. This resulted in drugs and/or alcohol use to deal with fears and emotions associated with sex, or ongoing avoidance of sex and forming romantic connections.

#### Theme 3.1: Changing relationship with drugs and/or alcohol

While some participants described casual drug use which did not lead to ongoing difficulties, other participants described a negative change in their relationship with alcohol and other drugs after acquiring HIV. They reflected that drugs offered an escape from internal turmoil related to low mood, fear and anxiety associated with having HIV. Many participants reported being ‘*stuck in a vicious cycle*’ involving sex with drug use and mental instability/low mood.

*“I felt so disgusting that I couldn’t bring myself to be intimate with [sexual partner named] and I ended up going off the rails after that. I started using a lot of chemical enhancers, so a lot of MDMA, speed and cocaine and went off the rails a bit.”* ID: 031, female, DX: mid 90s.

*“I had become depressed and the more drugs I was using [to have sex], the more depressed I was becoming. That’s the cycle of taking drugs and having crazy sex to make yourself feel better and you feel good for a while. The reverse is that you feel even worse about yourself and more depressed*.” ID: 021, male, DX: early 10s.

Participants who later stopped using drugs with sex described a long process of learning to have satisfying sex without chemical enhancements. Conversely, a participant who was sexually inactive at the time of interview attributed his lack of sex to no longer using drugs.

“*Trying to rediscover sex without drugs, because that's how it was for a long time. Then I just wouldn't have sex for a long while, because I thought drugs had to be involved, or I feared sex without drugs … Well, not so satisfied because I'm trying to just have sex without drugs and I'm trying to reteach myself. I'm not hugely satisfied at this stage, but [I’m heading] in the right direction.” ID: 002, male, DX: early 10s.*

*“When I stopped using all drugs … that’s when the sex stopped, too … if I didn’t have the drugs I didn’t have the sex. Since I have been drug-free I’ve been celibate.*” ID: 005, male, DX: late 80s.

#### Theme 3.2: Ongoing sexual inactivity and sexual difficulties

Some participants had reached a point of no sexual activity, or not seeking sexual or romantic relationships. They attributed this to ongoing fears of rejection by a sexual partner.

*“I think it’s all mental really … I don’t put myself out there. There’s a real fear of rejection and openness because of the need to disclose. There has been rejection and all those things before and that feeds back into not seeking any sort of sexual activity with anyone.”* ID: 041, male, DX: mid 00s

*“I thought if I did meet someone in the future I think I’d rather it was someone that was positive as well, so I didn’t have to go through that, I’ve already been through a lot of rejection, I don’t think I could face any more.* ID:046, female, DX: mid 00s.

### A model of sexual adjustment to HIV: the process, facilitators and barriers

Once the shock of diagnosis had subsided, participants continued to experience fears of rejection by a sexual partner and/or of transmission of HIV to another person. The presence, absence and combination of these fears influenced initial sexual behaviour post-diagnosis. This resulted in: a period of sexual inactivity for most, no interruption to sex life for some, and increased frequency of sex for others.

A number of facilitators of adjustment emerged. A partner’s accurate perception of risk of transmission, and acceptance of HIV as part of that person, were powerful in assisting the HIV-positive person to overcome fears of transmission and of rejection. Serosorting offered many a way of re-engaging with sex life while avoiding fear of rejection or fear of transmission. Some participants then later transitioned to having sex with HIV-negative partners. A number of males reported that by serosorting and being now free of fear of acquiring HIV, their frequency of sex increased and boundaries of sexual practice expanded. Engagement with the HIV-positive community offered opportunities to find role models and observe others living well, which promoted self-acceptance and shared strength in the face of difficulties. Information about the negligible risk of transmission once viral load was undetectable and use of pre-exposure prophylaxis (PrEP) by sexual partners, had the potential to reduce fears of transmission and also fears of rejection. Similarly, when health care professionals focused on discussing sexual wellbeing rather than just sexual risk, this facilitated a return to a fulfilling sex life. For some participants, this was a return to pre-diagnosis sex life and for others, this involved ongoing serosorting and/or less frequent sex.

Conversely, sexual adjustment was impeded when there was no partner, a partner denied HIV was part of that person, or a partner was unduly fearful of transmission. Similarly, lack of support from peer or community, or the belief that health professionals only cared about risk of transmission to others did not facilitate adjustment. Despite accurate knowledge of risk of transmission with UDVL, and increased use of PrEP, some participants continued to report ongoing sexual difficulties due to undue fear of transmission.

**Fig. 1:**
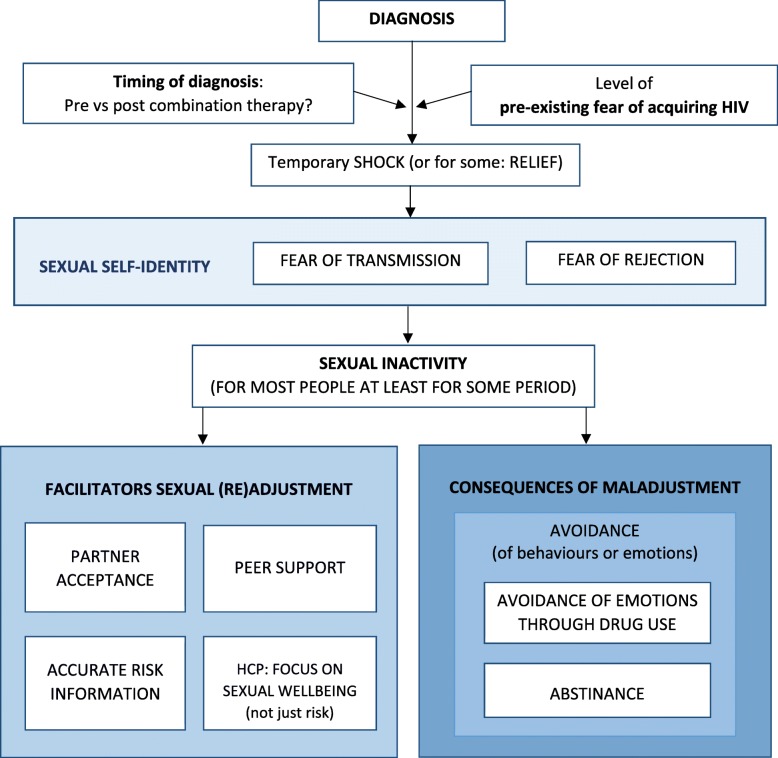
Depicts the diagrammatic model of the process of sexual adjustment to HIV resulting from this analysis

Persistent fears of transmission and fears of rejection seemed to be key factors associated with sexual activity, and so people with HIV reported strategies that helped them to avoid these negative emotions either by avoiding sexual activity itself, or by using substances that reduced the fears associated with sexual activity. As such, some participants reported increasing their use of non-prescribed drugs particularly in relation to sexual encounters, which some felt ultimately contributed to worse mental health outcomes and sexual-wellbeing (Fig. 1).

## Discussion

This study explored the process of sexual adjustment of PLWH over time. Fears of transmission during sex and rejection by sexual partners were central. Such fears determined initial sexual behaviour, and factors which assisted PLWH to overcome these negative emotions facilitated adjustment. Facilitators of adjustment included: partner acceptance, peer and community support, health professionals interested in sexual wellbeing (not just risk), and accurate knowledge of risk of transmission including of undetectable viral load (UDVL) and pre-exposure prophylaxis (PrEP).

Sexual inactivity and no interruption to sexual life as responses to an HIV diagnosis are consistent with past research findings [18–20]. However, feelings of relief, freedom from fear of acquiring HIV, an increase in the frequency of sex and expanding of sexual practice after diagnosis as potential responses to HIV diagnosis represent a novel contribution of the current study. Such responses to diagnosis have been previously documented in 1999, however, past research has previously conceptualised this as sexually deviant behaviour, labelled “bug chasing”, associated with the knowledge that acquiring HIV will cause death [21]. We conceptualise these responses in the current study as mainstream responses which reflect decreased fear of living with HIV in general since the introduction of combination therapy. Fear of transmission of HIV to a sexual partner has been mentioned as relevant to sexual difficulties in past research [18, 22–24], as has fear of rejection by a sexual partner [18, 25–27]. However, this study is the first to find that these fears determined initial sexual behaviour and were central to the process of adjustment over time, even when participants had accurate knowledge that could have refuted those fears.

A past study reported a single finding that supportive sexual partners were generally conducive to overcoming sexual difficulties [22]. The current study explored the role of partners in sexual adjustment to HIV in detail, and established the mechanism by which partners facilitate adjustment; namely, acceptance of HIV as part of shared sexual life, and demonstrating no undue fear of transmission of HIV. This study also makes a novel contribution about the role of peer and community to promote sexual adjustment through role modelling promoting self-acceptance, and highlights the mechanism by which health professionals can promote sexual adjustment, by demonstrating to their patients that they care about overall sexual wellbeing, not just sexual risk. Further research into partner directed information sharing and interventions, similar to those conducted in other health difficulties, is required [28].

It has been postulated that increasing knowledge of negligible risk of transmission of HIV with sustained undetectable viral load (U=U) will reduce fears of transmission and assist with overcoming HIV stigma [8]. To our knowledge, this is the first study to explore this assumption empirically. Of note, while many participants in this study reported that knowledge of U=U improved their sexual-wellbeing, some participants did not have this knowledge, and there were others who despite having this knowledge reported persistent sexual difficulties associated with fear of transmission. To ensure equal access to the benefits of the U=U message, further research is required to establish what proportion of PLWH do not have this knowledge, and what proportion have persistent sexual difficulties due to fear of transmission despite this knowledge. Such research will need to account for the difference between beliefs and knowledge, and the role of comprehension and health literacy in this context.

To facilitate sexual adjustment with HIV, the following recommendations for health professionals working with PLWH are proposed:

1. Directly intervene to assist partners of PLWH, particularly HIV-negative partners, to understand risk of transmission accurately to prevent undue fear.
2. Be aware that while serosorting behaviour can assist PLWH to re-engage with sexual life in the short to medium term, if it continues long-term it has the potential to perpetuate fears of transmission or rejection by giving PLWH the impression that they cannot have sex with HIV negative people safely.
3. Promote opportunities for positive connection between PLWH, including referral to peer led workshops and community groups
4. Assess sexual quality of life generally, not just sexual risk, and/or ensure that PLWH have access to psychosocial services (sexual health counselling) that can intervene to promote sexual wellbeing
5. Ask about knowledge of U=U, not simply being aware of the message generally, but ensure that PLWH understand how it applies to their sexual life, and sexual practices relevant to them (e.g. oral/vaginal/anal sex).

The strengths of this study lie in the rigorous application of theoretical sampling and constant comparison to achieve a novel process-based analysis with strong clinical applications. The findings of this analysis are limited to the experiences of PLWH in high-income nations with easy/unrestricted access to treatment and knowledge about risk of transmission. Self-selection bias may have caused under-representation of experiences of people less willing to discuss sexual life.

## Conclusions

This study is the first to explore the process of sexual adjustment to HIV over time, and the first to find that fears of transmission and fears of rejection are central to such adjustment. The model of sexual adjustment to HIV establishes a number of facilitators by which PLWH achieve such adjustment, including (a) partner acceptance of HIV within their sexual life, and no undue fear of transmission, (b) connections with peers, (c) health professionals who focus on sexual well-being not just risk, and (d) accurate knowledge of risk of transmission of HIV including accurate knowledge of UDVL and PrEP. By using this model of facilitators and barriers, health professionals can promote sexual adjustment and good overall sexual quality of life of PLWH.

## Data Availability

The datasets generated and/or analysed during the current study are not publicly available as verbatim interview transcripts even if de-identified could reveal the identity of the participant but are available from the corresponding author on reasonable request.

## Abbreviations

DASS-21: Depression Anxiety Stress Scales – 21item short form
PLWH: People living with HIV
PrEP: Preexposure prophylaxis
U=U: Undetectable = untransmittable
UDVL: Undetectable viral load
